# Effects of NH_4_^+^-N: NO_3_^−^-N ratio on growth, nutrient uptake and production of blueberry (*Vaccinium* spp.) under soilless culture

**DOI:** 10.3389/fpls.2024.1438811

**Published:** 2024-10-17

**Authors:** Ali Anwar, Junming Zheng, Chunfeng Chen, Mengqing Chen, Yanxu Xue, Jinmiao Wang, Wei Su, Riyuan Chen, Shiwei Song

**Affiliations:** College of Horticulture, South China Agricultural University, Guangzhou, China

**Keywords:** nitrogen, potassium, antioxidant, sugar, photosynthesis, blueberry

## Abstract

Blueberry (*Vaccinium corymbosum*) is a small pulp shrub, which prefers to grow on a soilless culture. For soilless culture, nutritional management remains typically vital for blueberry production. However, the effect of different nutritional treatments on blueberry growth and production is largely unknown. This study was designed to investigate to formulate a specific nutritional treatment for blueberry. The results showed that NH_4_^+^-N: NO_3_^−^-N ratios significantly affected the growth, nutrient uptake, physiological characteristics, and flowering, as well as the fruiting characteristics of blueberry plants. The number of shoots and top projection area was increased considerably by 25:75 treatment. In contrast, 50:50 treatment promotes plant height, shoot length, and stem thickness, increasing chlorophyll contents, photosynthetic capacity, and P, Ca, and Mg in leaves. In contrast, 50:50 treatment promotes the flowering fruiting rate and prolongs the blueberry flowering period. The maximum soluble sugar contents were noted in 25:75, while maximum starch contents were reported in the 50:50 treatment. The treatments 100:0 and 75:25 promote early flowering and accelerate fruit set. Notably, NH_4_^+^-N: NO_3_^−^-N ratios; 50:50 treatment significantly encourages plant growth, nutrient uptake, chlorophyll contents, photosynthetic capacity, and fruit setting rate in blueberry plants. These findings suggested that NH_4_^+^-N: NO_3_^−^-N ratios 50:50 is the most appropriate treatment that significantly promotes vegetative growth and enhances production in blueberry plants. This study provides valuable information for improved blueberry production under a controlled environment.

## 1 Introduction

Blueberry (*Vaccinium corymbosum*) is an important perennial shrub fruit plant belonging to the genus Vaccinium of the Ericaceae family (Han et al., 2021; Wu et al., 2022). It’s native to North America (Neugebauer et al., 2024; Wu et al., 2022) and because of its unique flavor and high nutritional value, its rapidly spreading to all over the world (Wu et al., 2022). The fruits of blueberry are oblate or spherical in shape, sift and juicier, having excellent levels of antioxidant active ingredients: polyphenols, anthocyanins, and flavonoids (Liu et al., 2022; Nagasaka et al., 2022). Blueberry fruit has prodigious nutritional and medicinal values, such as improving eyesight, aging, strengthening heart function, anti-cancer, softening blood vessels, and enhancing human immunity (Han et al., 2021; Stull et al., 2024). As the blueberry industry expands, the sustainability of blueberry is continuously threatened by environmental influences, that cause a significant reduction in production and quality (Tamir et al., 2023; Yang et al., 2023).

Blueberry is an oligotrophic plant, and the contents of nitrogen, phosphorus, potassium, calcium, and magnesium in the tree are shallow compared with other fruit trees (Shen et al., 2020; Ortiz-Delvasto et al., 2023; Yang et al., 2023; Ru et al., 2024). Blueberry is sensitive to fertilization, and improper fertilization will not only affect production but will even cause the leaves of blueberry branches to wilt and physiological dysfunction; in severe cases, it will lead to the whole plant falling and the branches withering and dying (Alt et al., 2017; Shen et al., 2020; Tang et al., 2024). Blueberries are primarily cultivated in soilless substrates culture, and fertilization and water supply are carried out simultaneously through the integrated system (Ortiz-Delvasto et al., 2023). For optimum plant growth, a suitable nutritional solution is required throughout the growth period. Nutrient solution management is a crucial part of soilless substrate cultivation technology. However, the formula composition, concentration, ratio of different nutrients, pH, and daily preparation of nutrient solution directly affect the absorption and utilization of nutrients by crop roots (Tamir et al., 2023). Therefore, these factors are significant for improved crop production and quality under soilless culture (Yang et al., 2022b; Tamir et al., 2023).

Typically, blueberries are cultivated through soilless culture, but up to now, the relevant research on the nutrient solution formula of blueberries remains unclear. Many studies focused on the adaptability of blueberries to nitrogen forms, the effects of nutrient deficiency, and the formulations of different nutrient solutions (Alt et al., 2017; Shen et al., 2020; Yang et al., 2022b; Wu et al., 2024). However, a few reports on the systematic research on the integrated nutrient solution formula of blueberry with water and fertilizer. For the sustainable development of the blueberry industry, it is crucial to optimize and adjust the nutrient solution formula to obtain a nutrient solution formula that is more suitable for cultivating blueberries.

The substrate cultivation is generally called the nutrient substrate, which not only has the primary function of fixing and supporting plants but also has the role of regulating nutrient and water supply (Cocetta et al., 2012; Yang et al., 2022b; Hu et al., 2023). The physicochemical properties of the substrate are crucial for its effective utilization, as it can directly affect the environment in which plant roots grow, thus affecting the growth and development of plants and ultimately determining the yield and quality of fruits to a certain extent (Palencia et al., 2016; Ortiz-Delvasto et al., 2023; Rasool et al., 2024). Soilless substrate cultivation has significant advantages over soil cultivation, which can avoid continuous cropping obstacles, avoid the occurrence of soil-borne diseases, reduce root physiological diseases caused by aeration problems, and make more efficient use of water and fertilizer, increase the planting adaptability of plants, and enable plants to be planted in some soil environments where average growth cannot be achieved (Nunez et al., 2023; Ortiz-Delvasto et al., 2023; Rasool et al., 2024). Blueberry root distribution is shallow, and primarily, it has fine roots without root hairs and weak absorption capacity. Hence, blueberries prefer loose and airy acidic soils with high organic matter content, which have high requirements for a rhizosphere environment (Saad et al., 2020; Nunez et al., 2023; Ortiz-Delvasto et al., 2023).

For the specialized root of the blueberry, the peat and coir can provide a favorable environment and water retention to the rooting system, thus supreme substrates for blueberry cultivation (Cocetta et al., 2012; Alt et al., 2017). In addition, peat with good cation exchange capacity, low content of phytotoxic substances, and perlite, which has good stability and good air permeability and does not produce components that interfere with the balance of nutrient solution, are also one of the most widely used substrates in soilless culture (Shen et al., 2020; Xu et al., 2020; Lu et al., 2022; Yang et al., 2022b; Nunez et al., 2023). Soilless substrate formula has good substrate permeability and water and fertilizer retention ability, which can provide the best rhizosphere environment for blueberry growth and promote the growth and development of blueberry. This study was designed to investigate the nutrient solution formula suitable for blueberry substrate cultivation and then determine the effects of different N and K levels on the growth and adaptability of blueberries. This study aims to explore the optimal nutrient treatment to provide theoretical guidance and practical reference for high-yield, high-quality, and efficient facility substrate cultivation and production of blueberry.

## 2 Materials and methods

The experiment was carried out in the control growth chamber of the College of Horticulture, South China Agricultural University. The highbush blueberry seedlings of “Nangao Z9”, were used as experimental material. The cultivation substrate was prepared according to peat:cocofen:perlite = 1:1:1 (see **Supplementary Table S1** for physical and chemical properties), and planted in square cultivation pots with specifications of 26 cm× 26 cm× 30 cm (length × width × height). Each trial was divided into five treatments, each with 18 blueberry plants. After planting, drip irrigation (8 times a day, 100 ml each time) with water was used for three days. The pH was set to 4.8 (the pH value of the nutrient solution was adjusted by sulfuric acid), and the treatment was started after three days. Growth-related indexes were determined on the 30th, 60th, and 90th days of treatment, and destructive sampling analysis was performed on the 90th day of treatment.

### 2.1 Experimental I

#### 2.1.1 Effects of different nutrient solution formulations on blueberry

To investigate the effects of different nutrient treatments on blueberry, the three existing blueberry nutrient solution formulas and two standard nutrient solution formulas were selected, shown in **Table 1**. After the trial, the indicators were measured and analysed, and the formula with the best comprehensive index was chosen as the basic formula for further experiments. The contents of trace elements in each treatment are shown in **Supplementary Table S1**.

**Table 1:** Concentrations of each element in different nutrient solution formulations.

Treatment	mmol.L^-1^					
	N		P	K	Ca	Mg
	NH_4_^+^-N	NO_3_^−^-N				
T1	0	15.0	1.0	6.0	5.0	2.8
T2	1.3	16.0	1.3	8.0	4.0	2.0
T3	7.3	8.0	1.3	4.2	4.0	2.6
T4	3.5	12.7	0.8	3.5	4.6	2.9
T5	3.8	4.8	1.0	3.0	2.4	1.1

### 2.2 Experimental II

#### 2.2.1 Effects of different nitrogen and potassium levels on blueberry

In this experiment, the best treatment was selected from the previous, while the concentration of N or K elements was changed, as shown in **Table 2**. Moreover, the concentration of other elements remained unchanged. There were 5 treatments, while the contents N and K concentrations in CK remained unchanged. The content of trace elements in each treatment is shown in **Supplementary Table S1**, and the concentration of macro-elements is shown in **Table 3**.

**Table 2:** The different concentrations of nitrate and ammonium.

Treatment	mmol.L^-1^		
	NH_4_^+^-N: NO_3_^−^-N	N	
		NH_4_^+^-N	NO_3_^−^-N
T1	100:0	11.5	0
T2	75:25	8.63	2.88
T3	50:50	5.75	5.75
T4	25:75	2.88	8.63
T5	0:100	0	11.5

**Table 3:** Proportion and concentration of nitrogen and potassium at different nitrogen and potassium levels.

Treatment	mmol.L^-1^			
	N:K	N		K
		NH_4_^+^-N	NO_3_^−^-N	
LN	2.91:1	5.5	6.0	4.2
HN	4.37:1	9.1	10.0	4.2
CK	3.64:1	7.3	8.0	4.2
LK	4.64:1	7.3	8.0	3.15
HK	3.04:1	7.3	8.0	5.25

### 2.3 Experimental III

#### 2.3.1 Effects of different levels of NH_4_ and NO_3_ on blueberry plants

Based on the previous experiment, the excellent treatment was used for further modification to investigate the effect of different ammonium nitrate ratios on blueberries. Under the total nitrogen amount determined in experiment II, five different levels of ammonium nitrate ratio (NH_4_^+^-N/NO_3_-N) were designed, i.e., 100:0, 75:25, 50:50, 25:75, 0:100, where (NH4) 2SO4 and NO3-N provided NH4+-N was provided by Ca (NO_3_)_2_ and KNO_3_, as mentioned in **Table 2**. The dosage of trace elements in each treatment is shown in **Supplementary Table S1**, and the concentration of ammonium nitrate nitrogen is shown in **Table 2**.

### 2.4 Determination of plant morphological indicators

Nine blueberry plants with uniform growth were labelled in each treatment, and morphological indexes were measured on the 30th, 60th, and 90th days of treatment. Measure the stem thickness of blueberry shoots through a digital vernier calliper and the length of blueberry shoots were measured by measuring tape. The blueberry leaf picture and the whole blueberry picture were taken, and the leaf area and top projection area (cm^2^) of the blueberry were calculated using Image J (V1.8.0) software.

### 2.5 Determination of photosynthetic pigments in blueberry leaves

The contents of chlorophylls were determined on 5th-7th leaves of the basal branches from top to bottom were harvested. 0.2g leaf tissue were inserted into 95% ethanol solution and put in dark for 24h, and then chlorophyll a (Chl A), chlorophyll b (Chl B), and carotenoid was measured as previously described. The optical density was recorded at 665, 649 and 470nm using a spectrophotometer (Li et al., 2022; Xing et al., 2022). The calculation formula is as follows:

- Chlorophyll a concentration (mg/L) = 12.7× OD665-2.69×OD649
- Chlorophyll b concentration (mg/L) = 22.9× OD649-4.86×OD665
- Total chlorophyll concentration (mg/L) = 8.02×OD6655+20.20×OD649
- Carotenoid concentration (mg/L) = 4.7×OD447-0.27× total chlorophyll concentration
- Photosynthetic pigment content (mg/g) = (photosynthetic pigment concentration × extraction volume) / sample mass

### 2.6 Determination of photosynthetic characteristics of blueberry leaves

After 90 days of treatment, 10 plants were randomly selected for each treatment, and the light and parameters of basal functional leaves (top-down 5-7) were measured by LI-6400 portable photosynthetic instrument at 9:00~11:00 a.m. in sunny weather, including net photosynthetic rate (Pn), transpiration rate (E), stomatal conductance (Gs), and intercellular CO_2_ concentration (Ci) (Zhao et al., 2024).

### 2.7 Determination of physiological indexes of blueberry leaves

To investigate the physiological indexes of blueberry leaves, the 5th to 7th leaves were harvested. The SOD activity and malondialdehyde contents were determined by the nitrogen blue tetrazolium (NBT) photochemical reduction method and thiobarbituric acid (TBA) reaction method, respectively (Wu et al., 2022).

### 2.8 Determination of soluble sugar and starch contents

The contents of soluble sugar in blueberry leaves were determined by using previously describe method. Briefly, 1g fresh samples were grounded and rinsed with ddH2O2 and then the extractions were filtered and boiled for 30mints, and the tube and residue were flushed to the final volume (100 ml). 0.5ml extract solution and 1.5ml ddH2O2 were added to 25ml tube and gently mixed with 0.5ml anthrone-ethyl acetate and 5 ml concentrated sulfuric acid, then boiled for 1min. After cooldown at room temperate, the absorbance was measure at 630nm (Wang et al., 2022).

Th contents of starch in blueberry leaves were determined by anthrone method. Briefly, 0.1g samples were homogenized in in 10ml of 80% of ethanol and then water bathed at 80°C for 30 mins. After centrifugation at 6000rmp for 10 mints, the supernatants were combined with 2ml on anthrone regent and biol again for 7mints. Finally, the absorbance was recorded at 620nm as described previously (Clegg, 1956).

### 2.9 Determination of mineral element contents in blueberry plants tissues

The leaves of blueberries (the third test were roots, stems, and leaves) were washed and dried and placed in an oven at 105°C for 30min, and dried to constant weight at 75°C to obtain dry samples. To determine the total nutrients contents samples were first digested in HNO_3_ by using a microwave digestion system (Mars X press Microwave Digestion system, CEM, Matthews, NC, USA), and then analysed by ICP-OES (ICP-OES, Optima 5300 DV, Perkin Elmer, Waltham, WA, USA) analyzer, while Jaldal Method was used for N contents determination (Huang et al., 2016; Rodríguez-Vila et al., 2022) The nutrient status of blueberry leaves in this experiment will be evaluated with reference to the previous grading standards for blueberry leaf nutrients (Huang et al., 2016).

### 2.10 Investigation of blueberry flowering and fruiting habits

From November 2022, the flowering rate was counted, and the first flowering stage, full flowering stage and last flowering stage of shoots were calculated, and 12 blueberry shoots were counted for each treatment. The petals are dehisced, revealing the stigma for which the flower opens. The flowering percentage reaches 20% for the first flowering stage, the flowering percentage reaches 50% for the full flowering stage, and the flowering percentage reaches 90% for the late flowering stage, and the flowering rate = number of flowers/total number of flowers ×100%. After pollination and fertilization, it can grow and develop normally to form young fruits, which is the success of fruit set, and the fruit setting rate = number of fruit sets/number of flowers × 100% (Yang et al., 2022b).

### 2.11 Data processing

In this experiment, SPSS 26.0 software was used for statistical analysis, and Duncan method was used for multiple comparisons of single factor tests, and *P*<0.05 reached a significant level of difference, and Origin 2021 software was used for plotting.

## 3 Results

### 3.1 The effects of different nutrient solution formulations on the growth form of blueberries

**Figure 1:**
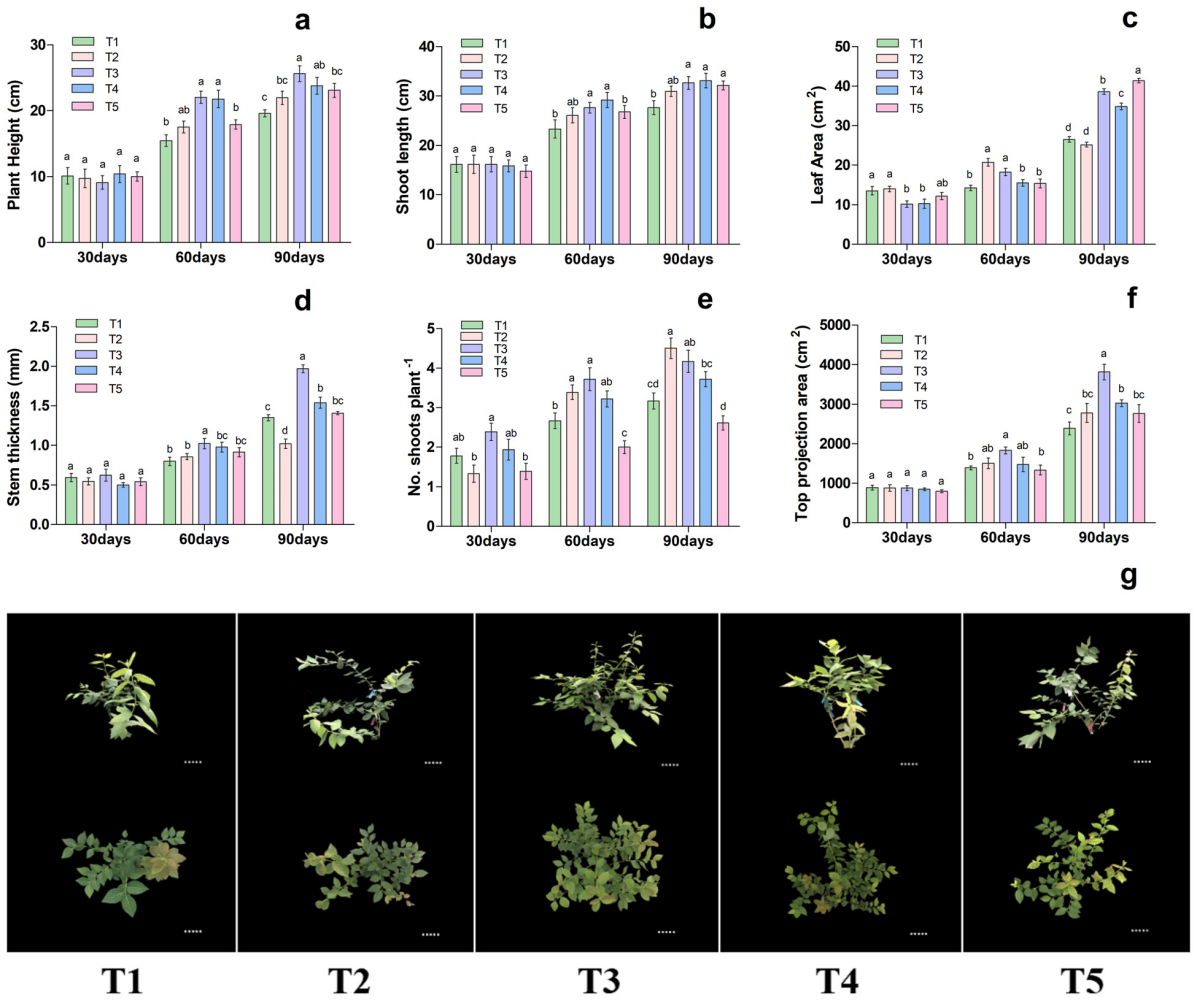
Changes in plant morphological characteristics under different nutritional treatments in blueberry. The growth morphology of the blueberry; **(A)** plant height, **(B)** shoot length, **(C)** leaf area, **(D)** stem thickness, **(E)** No od shoots plant^-1^, **(F)** top projection area, **(G)** blueberry shoot growth at different times 30days, 60days and 90days after treatments. The data indicated are the means ± SD. Different letters above the column indicate a significant difference between the treatments (*P*<0.05).

As shown in **Figure 1**. different nutritional treatments significantly affected on plant height, shoot length, leaf area, stem thickness, number of shoots plant^-1^, and top projection area of blueberry. The results showed that the plant height, shoot length, leaf area, stem thickness and top projection area on the 30th day among the treatments remained statically the same, whereas on 60 and 90th day of treatment showed significant differences as presented in **Figure 1**. In particular, the plant height, shoot length, leaf area, stem thickness, no. shoots plant^-1^ and top projection area were significantly increased by 42.60%, 18.34%, 28.05%, 28.21%, 39.58%% and 32.10% in T3 treatment respectively, when compared to T1. Likewise, on 90th day of treatment, the plant height, shoot length, leaf area, stem thickness, number of shoots plant^-1^ and top projection area were significantly increased by 30%, 18.06%, 45.51%, 46.47%, 31.57% and 59.81% were improved considerably in T3 treatment respectively, as compared to T1, as presented in **Figure 1**. Additionally, the different nutrient treatments potentially regulate the blueberry growth as presented in **Figure 1G**. These results suggested that T3 treatment was more conducive and significantly increased the blueberry plant growth, hence consider for a subsequent experiment.

### 3.2 Effects of different nitrogen and potassium levels on blueberry growth

**Figure 2:**
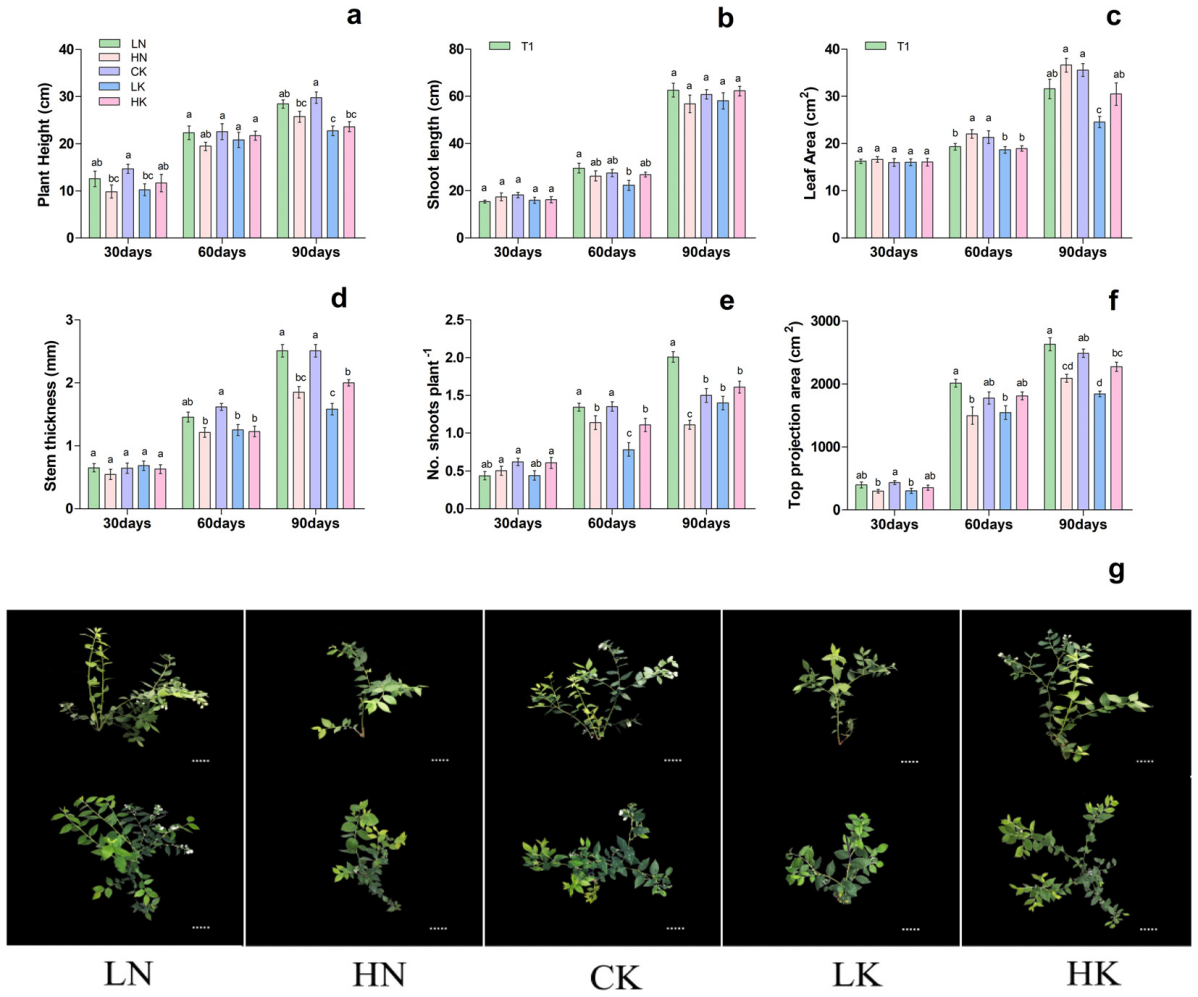
Changes in plant morphological characteristics under nitrogen and potassium levels (LN, HN, CK, LK, and HK) in blueberry. The growth morphology of the blueberry; **(A)** plant height, **(B)** shoot length, **(C)** leaf area, **(D)** stem thickness, **(E)** No od shoots plant^-1^, **(F)** top projection area, **(G)** blueberry shoot growth at different times 30days, 60days and 90days after treatments. The data indicated are the means ± SD. Different letters above the column indicate a significant difference between the treatments (*P*<0.05).

Based on previous experiments, treatment T3 was used to investigate the effect of different N and K levels on blueberry growth. As shown in **Figure 2**, different nitrogen and potassium levels significantly affected on blueberry plant height, shoot length, leaf area, stem thickness, no. of shoots plant^-1,^ and top projection area. The results showed that 30th day of treatment showed no significant difference in shoot length, stem thickness, and leaf area. In contrast, the top projection area in CK (control) and LK treatments was significantly lower than that of HN treatment, as presented in **Figure 2**. On the 60th and 90th days of treatment, the stem thickness and length were increased first and then decreased with the increase of nitrogen and potassium levels in the nutrient solution, respectively, and reported maximum under CK treatment. On the 60th and 90th days of treatment, the top projection area of plants decreased gradually with the increase of nitrogen level.

Meanwhile, with the rise in potassium level, the top projection area of the plant increased first and then decreased, reaching a maximum of 2489.34 cm^2^ in the CK. Likewise, the leaf area was increased gradually with the increase of nitrogen level, while with the rise in potassium levels, the leaf area was increased first and then decreased, and reported maximum at CK; 21.34 and 35.58 cm^2^, respectively (**Figure 2**). These results are predicted that, in comparison of CK and LN treatments, the blueberry plant growth of HN, LK and HK treatments was significantly reduced. Based on these findings, it can be concluded that LN and CK treatments were more favorable for the growth of blueberries.

### 3.3 Effect of different proportions of ammonium nitrate nitrogen on blueberry growth

**Figure 3:**
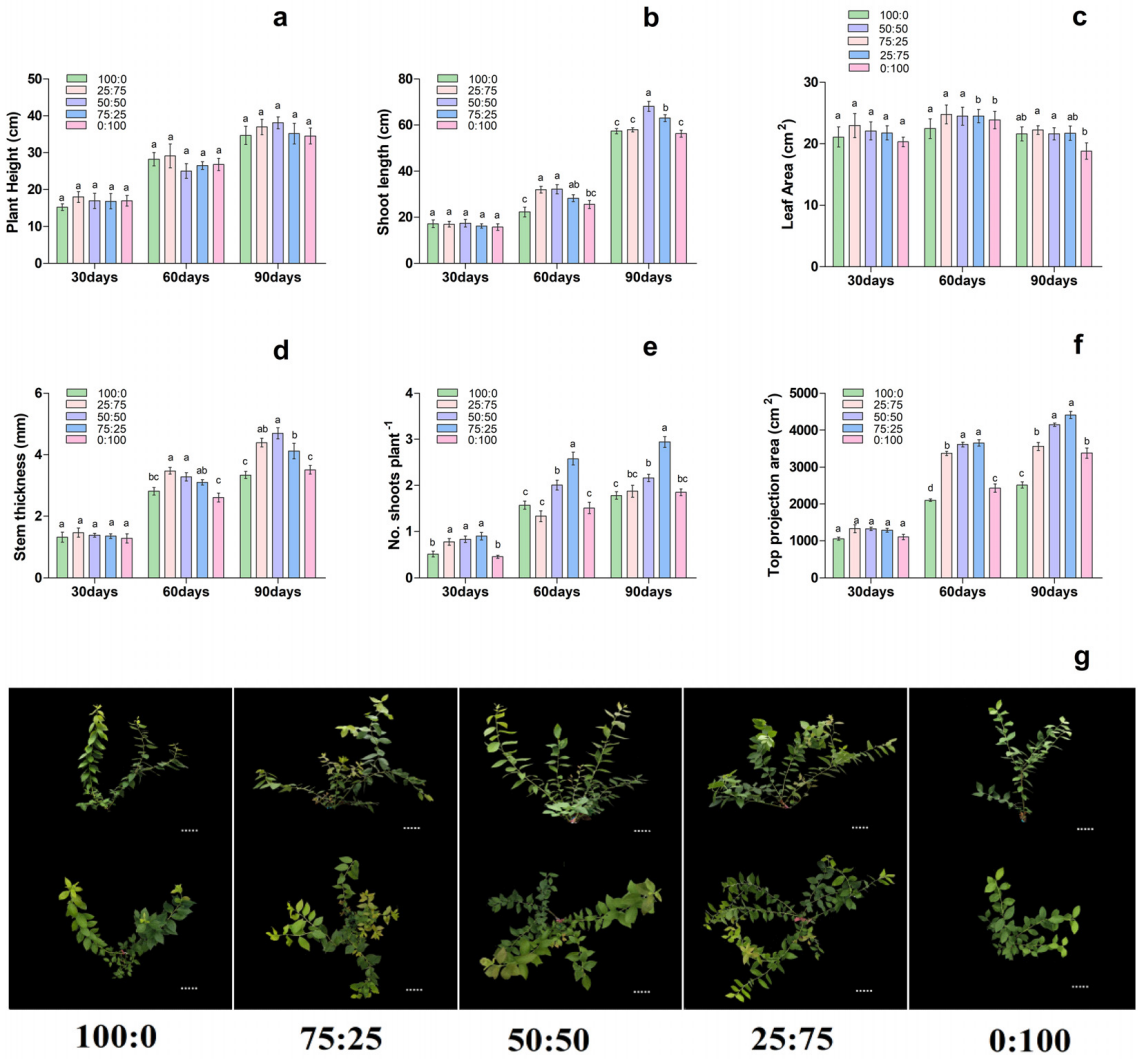
Changes in plant morphological characteristics under nitrogen and potassium levels (LN, HN, CK, LK and HK) in blueberry. Ther growth morphology of the blueberry; **(A)** plant height, **(B)** shoot length, **(C)** leaf area, **(D)** stem thickness, **(E)** No od shoots plant^-1^, **(F)** top projection area, **(G)** blueberry shoot growth at different times 30days, 60days, and 90days after treatments. The data indicated are the means ± SD. Different letters above the column indicate a significant difference between the treatments (*p*<0.05).

To further investigate the effect of proportions of ammonium (NH_4_^+^) and nitrate (NO_3_^-^) (NH_4_^+^-N: NO_3_^−^-N) on blueberry, then premium treatment LN was modified through NH_4_^+^ and NO_3_^-^ levels. The results showed that, with the increase of treatment time, the effect of different proportions of NH_4_^+^ and NO_3_ levels on the growth morphology of blueberry plants gradually increased, thus significantly affecting the growth morphology of blueberry seedlings (**Figure 3**). On 30th day of treatment, there were no significant differences in stem thickness, stem length, top projection area and leaf area of each treatment. However, on 60th day of treatment, with the decrease of the ratio of NH_4_^+^-N: NO_3_^−^-N, the stem diameter growth of the extended branches of the plants increased first and then decreased, and the 75:25 and 50:50 treatments were significantly higher than those of NH_4_^+^; 100:0 and NO_3_; 0:100 treatments. The top projection area of plants also increased first and then decreased with the decrease of the ratio of NH_4_^+^-N: NO_3_^−^-N, and the 50:50 and 25:75 treatments were significantly higher than those of the other treatments. Moreover, the plant height, stem diameter growth, leaf area, and top projection area of the plants increased first. Then it decreased with the decrease of the ratio of NH_4_^+^-N: NO_3_^−^-N, reaching the maximum at 50:50 and 25:75 treatments, respectively as presented in **Figure 3**. In conclusion, on the 90th day of treatment, the growth of plants increased first and then decreased with the decrease of the ratio of NH_4_^+^-N: NO_3_^−^-N, and compared with the single form of nitrogen, the mixed application of ammonium and nitrate was significantly more conducive to the growth of plants.

#### 3.3.1 Effect of different proportions of NH_4_^+^-N: NO_3_^−^-N, on photosynthetic pigments in blueberry leaves

**Figure 4:**
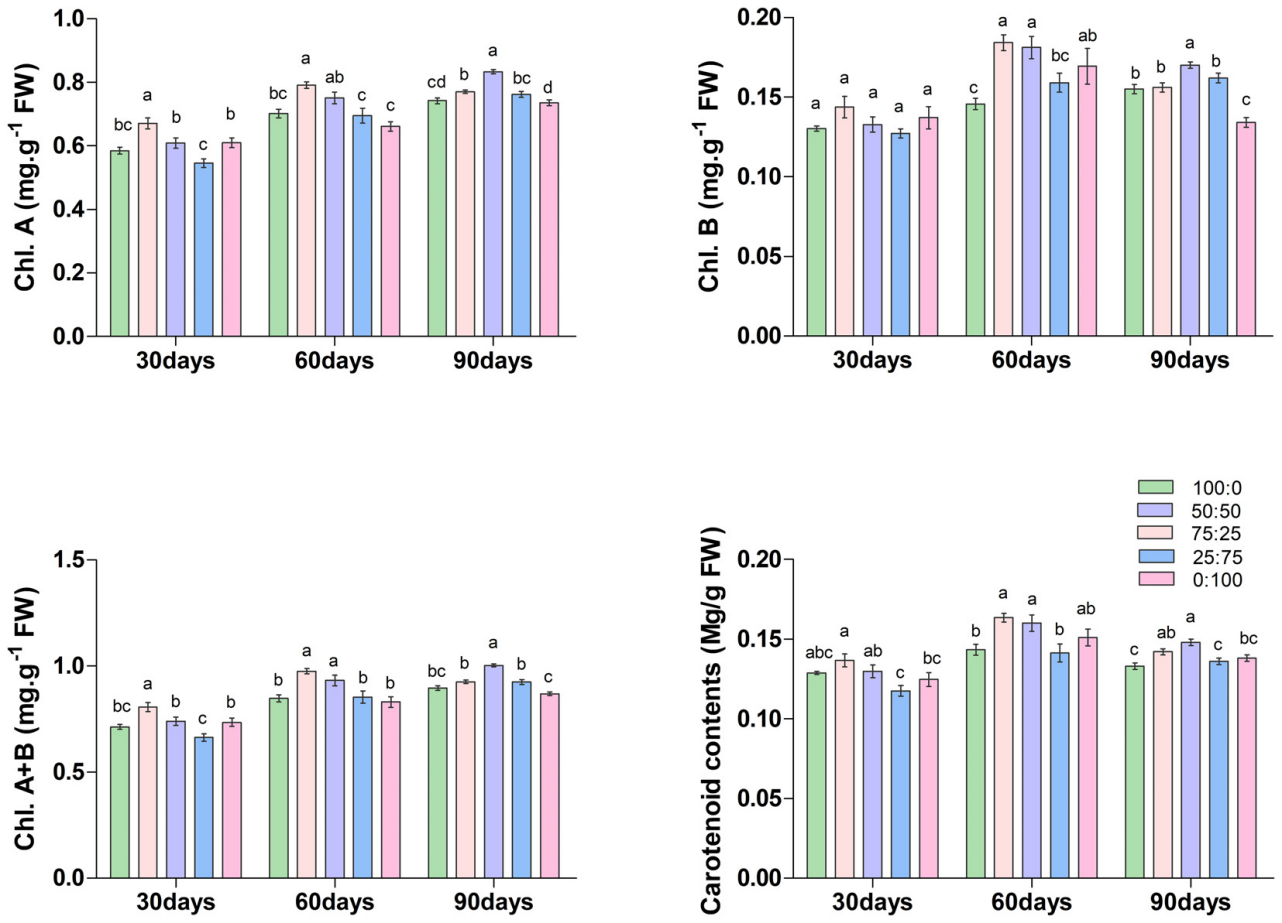
The effect of NH_4_^+^-N: NO_3_^−^-N levels on chlorophyll content in blueberry. Results are presented as the mean (n=4) and the bars above columns represent the standard deviation (SD). Different letters above the bars indicate significant differences (*P*< 0.05).

Chlorophyll is the most crucial pigment that plays an essential role in plant growth and development; thus considered a key growth indicator. The results of this study showed that the ratios of NH_4_^+^-N: NO_3_^−^-N caused a significant variation on the accumulation of chlorophyll and carotenoid contents in blueberry (**Figure 4**). On the 30th and 60th days of treatment, the contents of chlorophyll a (Chl. A), chlorophyll b (Chl. B), total chlorophyll (Chl. A+B) and carotenoids increased first and then decreased with the decrease of the ratio of NH_4_^+^-N: NO_3_^−^-N, and all reached the maximum at 75:25 treatment. Whereas, at 90th day of treatment, the content of each chlorophyll was increased first and then decreased with the decrease of the ratio of NH_4_^+^-N: NO_3_^−^-N, but reached the maximum value at 50:50 treatment. These results determine that the application of a higher proportion (75:25) of ammonium/nitrate in the early and middle stages of treatment would promote the accumulation of photosynthetic pigment in blueberry leaves. However, with the increase of treatment time, the application of an appropriate ratio (50:50) of ammonium nitrate nitrogen could promote the accumulation of photosynthetic pigment in leaves. Their findings suggest that the composition NH_4_^+^-N: NO_3_^−^-N ratio played a crucial role in chlorophyll biosynthesis in blueberry.

#### 3.3.2 Effect of different proportions of ammonium nitrate nitrogen on photosynthetic parameters of blueberry leaves

**Figure 5:**
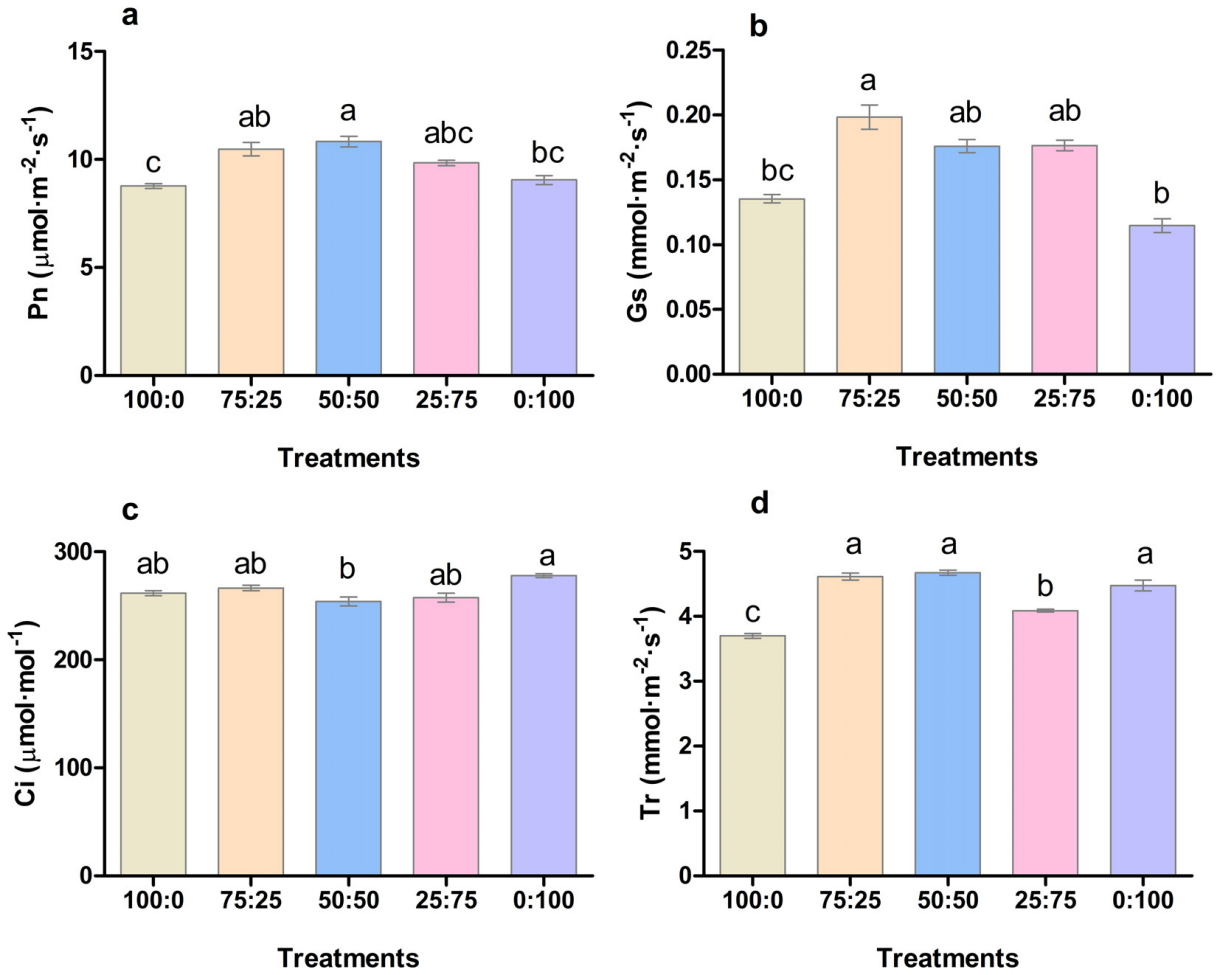
The effect of NH_4_^+^-N: NO_3_^−^-N levels on photosynthesis **(A)**; net photosynthetic rate (Pn), **(B)**; stomatal conductance (Gs), **(C)**; intercellular CO2 concentration (Ci), and **(D)**; transpiration rate (Tr) in blueberry. Results are presented as the mean (n=4), and the bars above columns represent the standard deviation (SD). Different letters above the bars indicate significant differences (P< 0.05).

The application of different proportions of NH_4_^+^-N: NO_3_^−^-N had a significant effect on the photosynthetic capacity of blueberry leaves. As presented in **Figure 5**, the net photosynthetic rate (Pn), transpiration rate (Tr), and stomatal conductance (Gs) of blueberry leaves increased first. Then, it decreased with the decrease of the ratio of NH_4_^+^-N: NO_3_^−^-N. The Pn rate and Tr reaching the maximum at 50:50, whereas Gs was noted maximum at 50:50, followed by 75:50 treatment as presented in **Figure 5D**. Similarly, intercellular concentration CO_2_ (Ci) of blueberry leaves decreased first and then increased with the decrease of the ratio of NH_4_^+^-N: NO_3_^−^-N, and was the lowest was reported in 50:50 treatment. The results indicated that the application of ammonium nitrate nitrogen in an appropriate ratio (50:50) was more conducive to the photosynthesis of blueberry leaves.

#### 3.3.3 Effects of different proportions of NH_4_^+^-N: NO_3_^−^-N on SOD activity and MDA content in blueberry leaves

**Figure 6:**
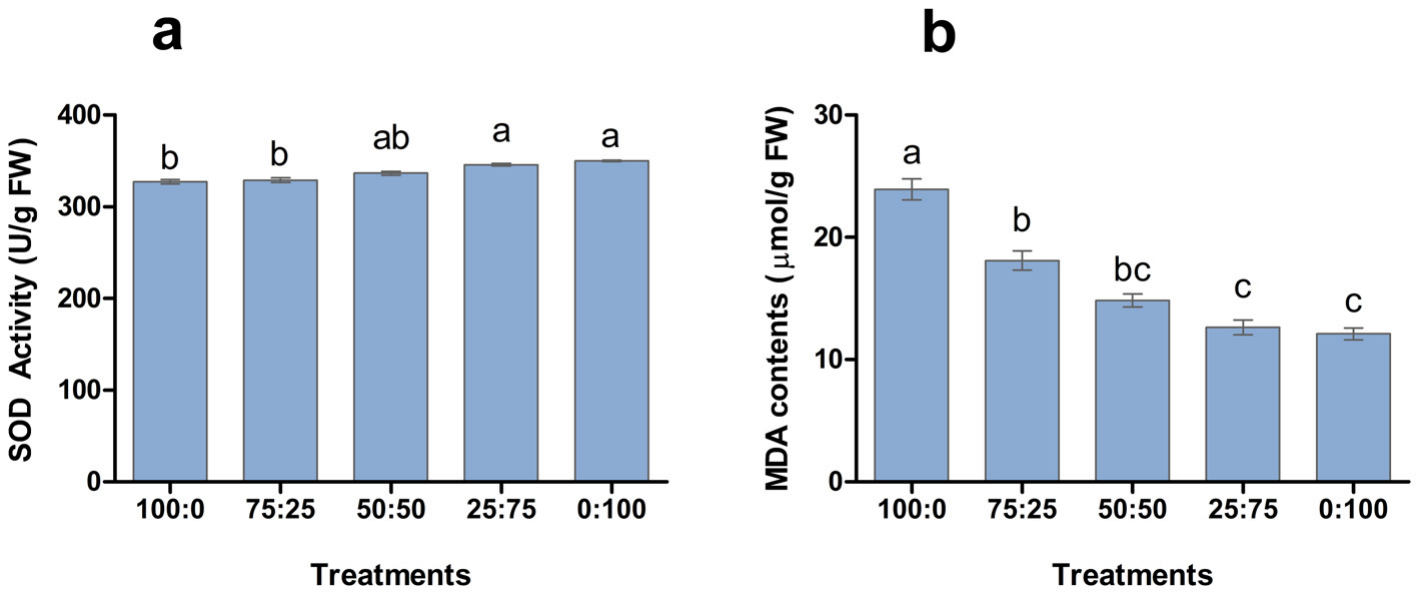
The effect of NH_4_^+^-N: NO_3_^−^-N levels on SOD activity **(A)** and MDA contents **(B)** in blueberry. Results are presented as the mean (n=4) and the bars above columns represent the standard deviation (SD). Different letters above the bars indicate significant differences (P< 0.05).

Antioxidant enzymes play a crucial role in plant response to environmental hurdles and reduce the harmful effects. The involvement of NH_4_^+^-N: NO_3_^−^-N in SOD enzyme activity and MDA production was analysed in blueberry leave (**Figure 5**). The results showed that SOD activity and MDA contents of blueberry leaves changed regularly with the decrease of the ratio of NH_4_^+^-N: NO_3_^−^-N. The higher the proportion of nitrate application enhanced the SOD activity of leaves and reported the maximum 1:100 treatment. However, the higher proportion of ammonium application resulted in a significant increment in MDA contents, as shown **Figure 6**. The maximum MDA contents were noted in the whole ammonium treatment 100:0. These results suggest that increasing the proportion of nitrate in the nutrient solution is beneficial to reduce the stress on blueberry plants.

#### 3.3.4 Effect of different proportions of ammonium nitrate nitrogen on the mineral element content of blueberry leaves

**Figure 7:**
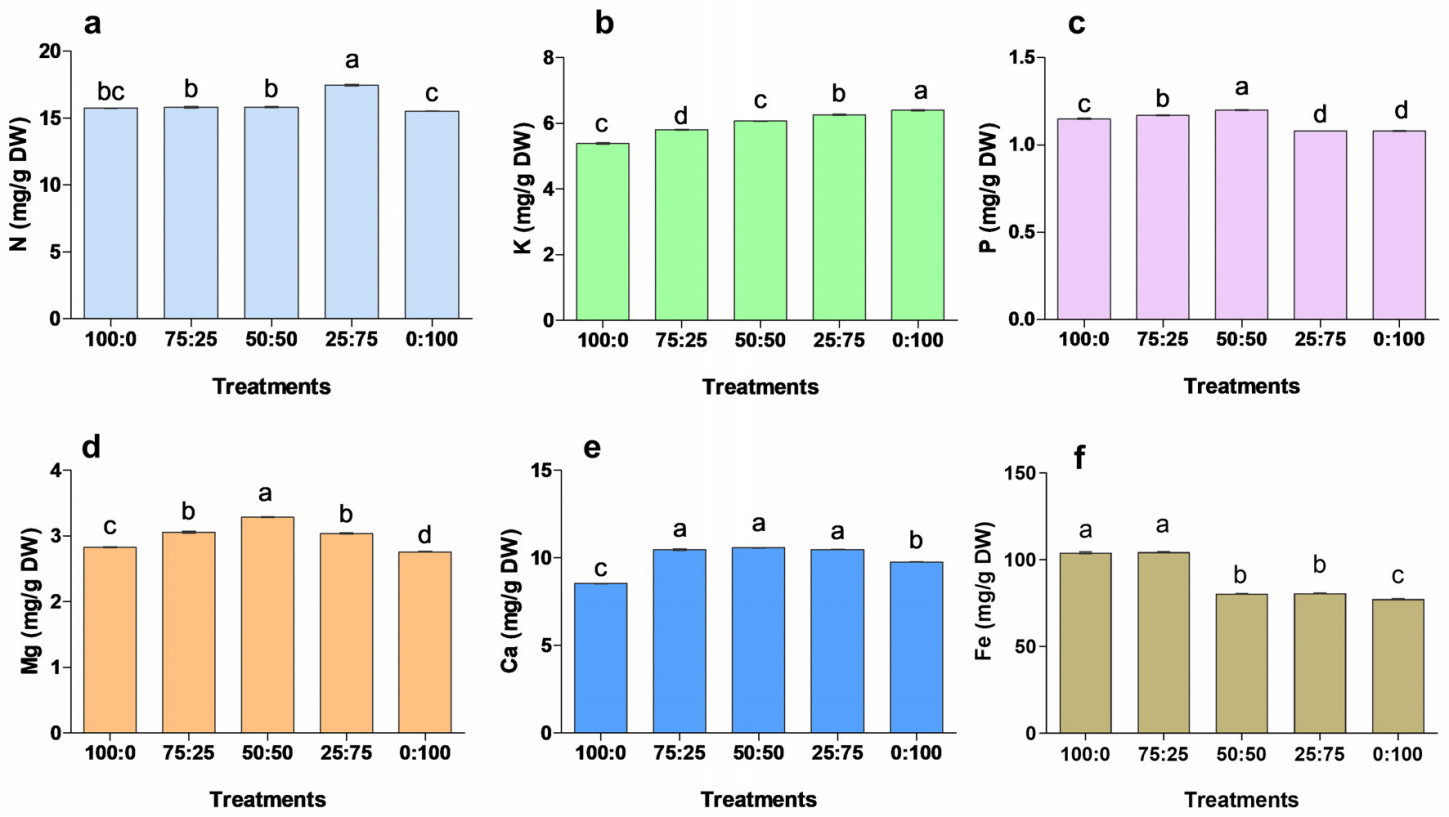
The effect of NH_4_^+^-N: NO_3_^−^-N levels on nutrient accumulation in blueberry **(A–F)**. Results are presented as the mean (n=4) and the bars above columns represent the standard deviation (SD). Different letters above the bars indicate significant differences (P< 0.05).

After 90 days of treatment, the application of different proportions of NH_4_^+^-N: NO_3_^−^-N had a significant effect on the mineral accumulation of blueberry leaves (**Figure 7**). The contents of N, P, Ca and Mg in blueberry leaves were increased first and then decreased with the increase of nitrate nitrogen application. The N contents were significantly maximum (17.46 mg/g) in 25:75 treatment. In contrast, P, Ma and Ca contents were 1.20 mg/g, 10.58 mg/g, and 3.29 mg/g in 50:50 treatment, respectively, were significantly higher when compared to other treatments. The K contents were considerably higher in 0:100 treatment, while the minimum K was noted in 100:0 treatment. The results showed that K contents were gradually increased with the increase of N contents. While, the Fe contents were significantly higher in 100:0 and 75;25 treatments, whereas minimum Fe was reported in 0;100 treatments. These findings suggest that NH_4_^+^-N: NO_3_^−^-N ratio was key in nutrient uptake and accumulation, thus improving blueberry growth and production.

#### 3.3.5 Effect of different proportions NH_4_^+^-N: NO_3_^−^-N on soluble sugar and starch

**Figure 8:**
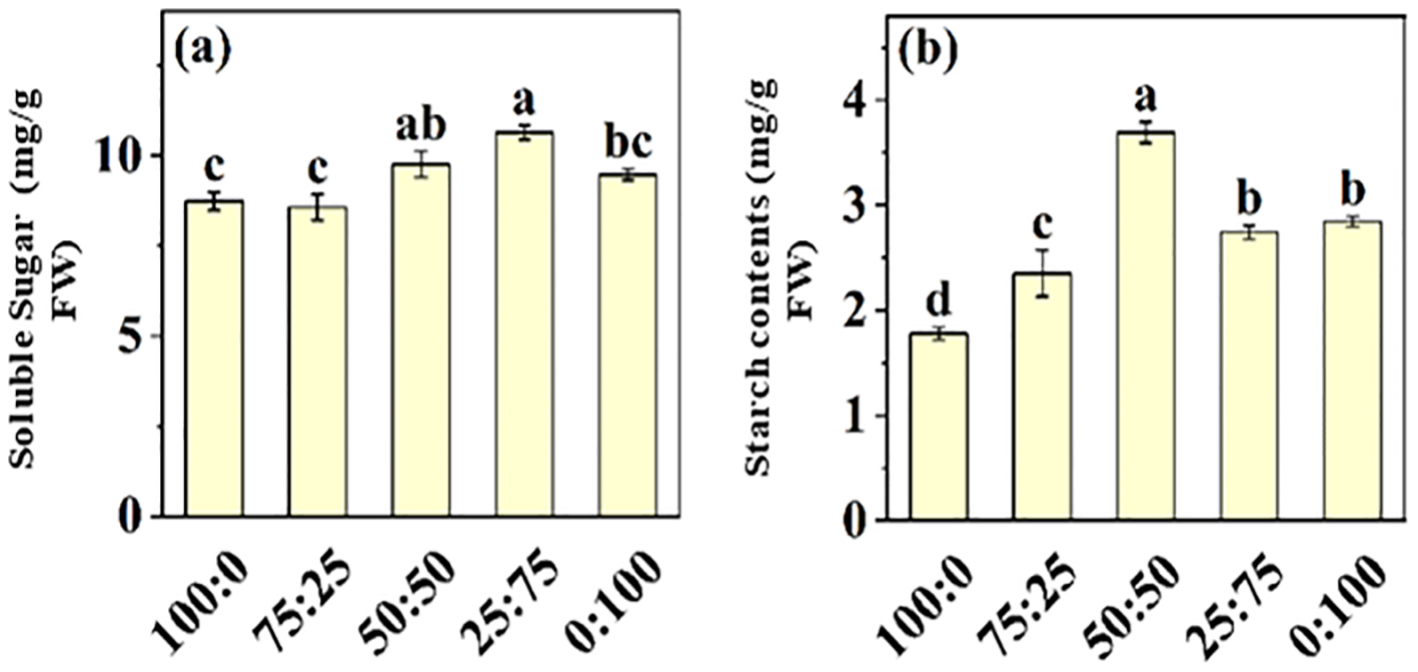
The effect of NH_4_^+^-N: NO_3_^−^-N levels on soluble sugar **(A)** and starch contents **(B)** in blueberry. Results are presented as the mean (n=4) and the bars above columns represent the standard deviation (SD). Different letters above the bars indicate significant differences (*P*< 0.05).

Nitrogen is the essential macro-nutrient for plant growth and developmental processes, involved in synthesizing amino acids, proteins, carbohydrates, proteins, chlorophyll, and other metabolites. Here, we investigate the effect of NH_4_^+^-N: NO_3_^−^-N ratio on soluble sugar and starch contents in blueberry leaves, as presented in **Figure 8**. The results showed that soluble sugar and starch contents of blueberry leaves increased first and then decreased with the increase of NH_4_^+^-N: NO_3_^−^-N ratio. The maximum soluble sugar content (10.64 mg/g), which was 21.88% higher than that of the all-ammonium treatment, was noted in 25:75 treatment. These results predicted that the application of NH_4_^+^-N: NO_3_^−^-N ratio in an appropriate ratio (50:50) was more conducive to the occurrence of carbon and nitrogen metabolism in blueberry leaves, hence improving blueberry growth and production.

#### 3.3.6 Effects of different proportions of ammonium nitrate nitrogen treatment on blueberry flowering

**Figure 9:**
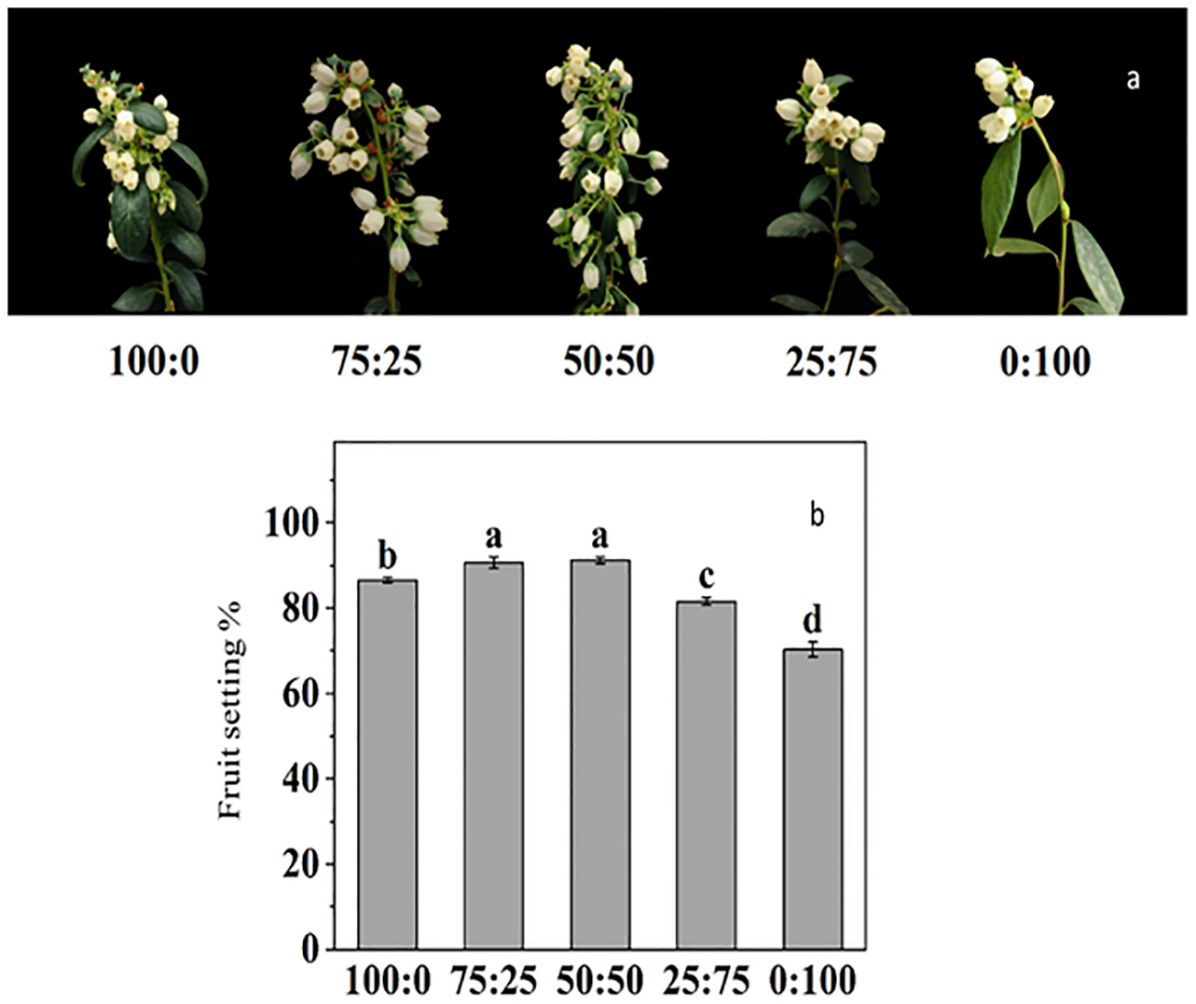
The effect of NH_4_^+^-N: NO_3_^−^-N levels on flower induction **(A)** and fruit setting percentage **(B)** in blueberry. Results are presented as the mean (n=4) and the bars above columns represent the standard deviation (SD). Different letters above the bars indicate significant differences (p< 0.05).

For the blueberry plant, flower development, initiation, and inductions are primarily critical, due to its high sensitivity to environmental cues. Here in this study, we reported that NH_4_^+^-N: NO_3_^−^-N ratio significantly affected on reproductive growth (**Figure 9**). The results showed that there were differences in the timing, such as first flowering, full flowering and late flowering of blueberries with different NH_4_^+^-N: NO_3_^−^-N treatment. The 50:50 treatment was the first to enter the early flowering stage, which was four days earlier than the total ammonium (100:0) and 15 days earlier than the total nitrate treatment (0:100). However, 100:0 treatment entered the complete blooming stage significantly earlier than other treatments, which was 2, 3, 6 and 13 days earlier than the other four treatments, respectively. In contrast, the full flowering stage was also delayed with an increased NH_4_^+^-N: NO_3_^−^-N ratio.

The fruit setting rate of blueberry was significantly affected by NH_4_^+^-N: NO_3_^−^-N treatments. As presented in **Figure 9B**, the increase in the proportion of NH_4_^+^-N: NO_3_^−^-N, the fruit setting rate of blueberry, increased first and then decreased. The maximum fruit-setting rate reported 50:50 treatment reached 91.23%, but there was no difference with 75:25 treatment, while the lowest fruit-setting rate 70.28%, was reported in NO_3_ (0:100) treatment.

#### 3.3.7 Correlation analysis and principal component analysis and comprehensive evaluation of blueberries treated with ammonium nitrate nitrogen in different proportions

**Figure 10:**
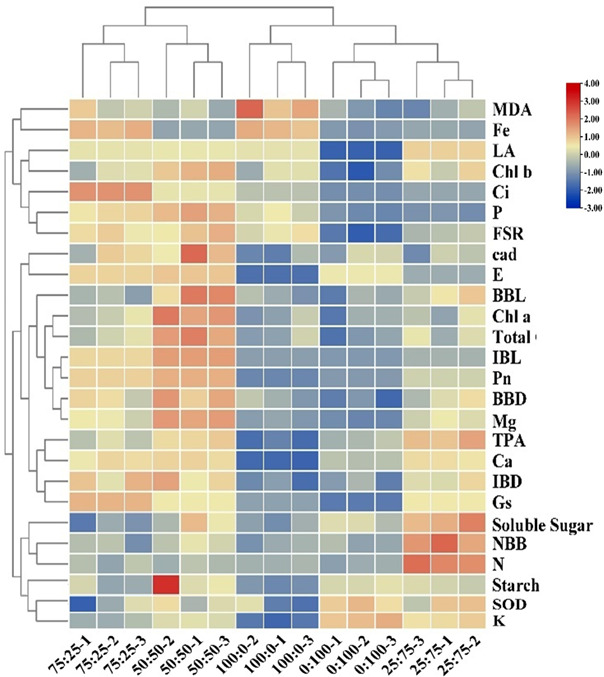
Cluster analysis of blueberry indexes in different proportions of NH_4_^+^-N: NO_3_^−^-N treatments.

**Figure 11:**
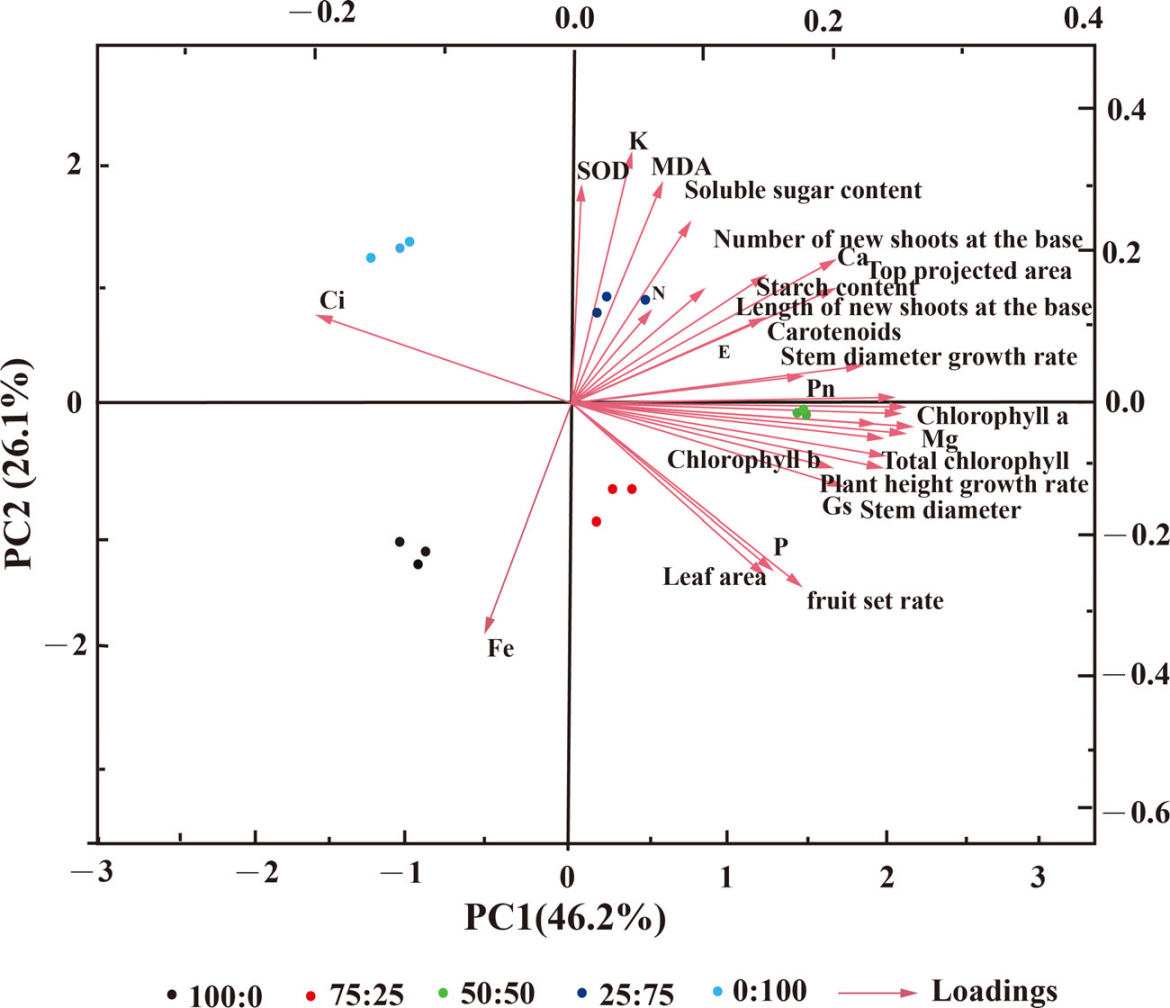
Principal component analysis (PCA) diagram of blueberry treated with NH_4_^+^-N: NO_3_^−^-N in different proportions.

To further investigate and clarify the effect of nitrogen and potassium levels on blueberry growth, the correlation and PCA analysis were carried out as presented in **Figures 10**, **11**, respectively. The correlation analysis suggested that the 100:0 treatment had high antioxidant enzyme activity in ammonium cations, while 75:25 and 50:50 treatments could significantly promote plant growth, chlorophyll contents, and photosynthesis. The 25:75 treatment was conducive to promoting the germination of blueberry basal growth branches and the accumulation of chlorophyll (**Figure 10**). These results suggest that 50:50 NH_4_^+^-N: NO_3_^−^-N level resulted in a significant improvement in blueberry growth and production.

However, the PCA analysis indicated that the cumulative contribution rate is greater than 85%, and the cumulative variance contribution rate of the first four principal components was 91.07%, which contained information on blueberry growth and development. The positive effects on the first principal component are mainly chlorophyll, the growth, branch length, the growth of basal branches, the net photosynthetic rate, and other indicators, and the loading weight is significant. The adverse effects are mainly Fe and intercellular carbon dioxide concentration (**Figure 11**). The key indexes such as SOD, MDA, soluble sugar, and K content had a positive effect on the second principal component. They had a considerable load weight, among which the leaf area, fruit setting rate, and P content had adverse effects. The distribution between the five treatments was far, indicating a significant difference. These results suggest that NH_4_^+^-N: NO_3_^−^-N ratio significantly affected blueberry growth and production.

## 4 Discussion

N is one of the essential macronutrients that play a role in plant growth and development, as well as crop production (Ye et al., 2022; Fu et al., 2023). Nitrate act as a signalling molecule that directly regulates the expression of key genes involved in regulating root and leaf development, cells expansion, regulating gene expression, and metabolism enzymes (Manoli et al., 2016; Tarkowski et al., 2024). The availability of N in the soil is primarily limited, while the application of additional N fertilizer to improve high crop production which not only increases input cost but also causes soil pollution (Kicklighter et al., 2019; Carillo and Rouphael, 2022; Vitousek et al., 2024). N uptake and accumulation by plants from soil in two forms: ammonium (NH_4_^+^) and nitrate (NO_3_^-^). In plants, the metabolism of N is followed by three key steps, including ammonia and nitrate transport, nitrate reduction and ammonia assimilation (The et al., 2021). Nitrate and ammonium transporters (low and high-affinity transporters) are involved in the absorption and transportation of NH_4_^+^ and NO_3_^-^ ions on the root surface to the rest of plant tissues (Tarkowski et al., 2024). After absorption, nitrate ions are reduced into nitrite by nitrate reductase (NR) in cytoplasm and then transported into plastid, where it can be reduced into ammonium through nitrite reductase (NiR) enzyme (Tabatabaei et al., 2008; Zhao and Shen, 2018; Morales De Los Ríos et al., 2021). Therefore, the appropriate ratio of NH_4_^+^ */*NO_3_^-^ might be substantial for plant growth and development by enhancing the uptake rate and accumulation of N in plant tissues (Tabatabaei et al., 2008; Zhu et al., 2021). Previous studies reported that appropriate NH_4_^+^ */*NO_3_^-^ ratio are important for chlorophyll synthesis, nutrient uptake and optimum plant growth and biomass in rice and Chinese cabbage (Tabatabaei et al., 2008; Zhu et al., 2021; Fu et al., 2023). Moreover, the NH_4_^+^ */*NO_3_^-^ ratio for plants varies from plant to plant and species. Likewise, 25:75 NH_4_^+^ */*NO_3_^-^ treatment for tomato, strawberry, and Chinese cabbage (Tabatabaei et al., 2008; Zhu et al., 2021; Gholamnejad et al., 2023)., while 0;100 for wheat and 50:50 is the optimal ratio for soybean (Raza et al., 2021; Yang et al., 2022a).

Herein, in this study the blueberry plant height, shoot length, leaf area, and stem thickness were significantly increased in 50:50 NH_4_^+^/NO_3_^-^ ratio, followed by 75:25 treatment (**Figure 3**). Moreover, the application of minimum NH_4_^+^/NO_3_^-^ ratio (25:75) promotes the blueberry branches, top projection area, and improve the lateral growth of the plants. However, the maximum plant height, stem length, and diameter of blueberry branches were noted in 50:50 NH_4_^+^/NO_3_^-^ ratio, thus indicating an appropriate ammonium-nitrate ratio (25:75~50:50) may promote the growth of blueberry basal branches (**Figure 3**). Previous study reported that, under the specific condition of a total nitrogen concentration, the optimal NH_4_^+^/NO_3_^-^ ratio showed better growth characteristics and more significant biomass accumulation in Chinese cabbage seedlings, compared to the treatment with a single nitrogen source (Alt et al., 2017; Yang et al., 2023). Similarly, in this study, the 75:25, 50:50, and 25:75 treatments significantly increased blueberry growth compared to the full ammonium or total nitrate treatments. Likewise, the contents of chlorophyll (**Figure 4**) and photosynthetic capacity (**Figure 5**) were enhanced by 75:25, 50:50 treatments, respectively. These results indicate that NH_4_^+^ and NO_3_^-^ combinations maybe beneficial for synthesizing photosynthetic pigments in blueberry leaves and to improve production. Under a specific total nitrogen concentration, the chlorophyll content and net photosynthetic rate of blueberry leaves were increased compared with NH_4_^+^ and NO_3_^-^ alone (Tabatabaei et al., 2008; Alt et al., 2017). These results are supported by previous studies, which reported that the NH_4_^+^ and NO_3_^-^ treatment regulate plant growth, expression of candidate genes of photosynthesis machinery and chlorophyll metabolism in rice and tomato seedlings (Fu et al., 2023; Gholamnejad et al., 2023). Thus, it can be concluded that NH_4_^+^ and NO_3_^-^ treatment 50:50 is most beneficial and hence causes a significant increment in blueberry seedling growth through improving chlorophyll accumulation and photosynthesis (Hao et al., 2023).

The absorption and transportation of NH_4_^+^ and NO_3_^-^ ions in plants are mostly affected by various factors, including soil environment, abiotic stresses, crop species and genotype (Alt et al., 2017; Fu et al., 2023; Chen et al., 2024). Previous studies reported that crops suitable for acidic soils tend to prefer NH_4_^+^, while crops ideal for neutral or alkaline soil environments tend to show a preference for NO_3_^-^ fertilizers (Cui et al., 2017; Zhu et al., 2021), because in acidic soils with low pH, the nitrifying process cannot be carried out generally due to the decrease in nitrifying bacteria. The available nitrogen produced by microorganisms from decomposing organic matter is mainly ammonium nitrogen, while in neutral and alkaline soils, the available nitrogen produced by nitrifying bacteria is mainly nitrate nitrogen (Santos et al., 2018; Goñi et al., 2021). Soil pH mostly change after crops absorb NH_4_^+^ and NO_3_^-^ ions, affecting the availability of other nutrients. Hence, these two forms of nitrogen and other ions have a direct or indirect effect on plant growth (Cui et al., 2017). A previous study reported that the ratio of NH_4_^+^ and NO_3_^-^ had a different impact on the biomass accumulation of various crops. In tomato, the ratio of NH_4_^+^ and NO_3_^-^ treatment, 50:50 and 25:75 was most beneficial for the growth of tomato seedlings (Gholamnejad et al., 2023). Likewise, 75:25 NH_4_^+^ and NO_3_^-^ treatment significantly improve seedling growth and biomass in rice and maize (Wang et al., 2023; Yunchao et al., 2024). Transcriptomic analysis showed that 75:25 treatment regulates the expression of nitrogen metabolism, carbon fixation in the photosystem, photosynthesis, starch and sucrose metabolism, and zeatin synthesis (Wang et al., 2020; Wang et al., 2023). Thus, these findings suggest that the appropriate ratio of NH_4_^+^ and NO_3_^-^ is crucial primarily for plant growth and development.

Blueberry plants are susceptible to environmental cues, which lead to a significant decline in growth and production (Alt et al., 2017; Nunez et al., 2023). The uptake and distribution of essential nutrients are crucial for maintaining homeostasis and plant growth under abiotic stress (Sathee et al., 2021; Carillo and Rouphael, 2022). Abiotic stresses significantly reduced the mineral uptake and accumulation, ultimately causing growth and biomass in cucumber and tomato seedlings (Raj et al., 2021; Ye et al., 2022). Herein, this study the ratio of NH_4_^+^ and NO_3_^-^ treatments regulated the minerals uptake and accumulation in blueberry tissues **Figure 7**. Previous study showed that the mineral uptake and accumulation of P, K, Ca, Fe, Mn, and B were enhanced by 1:1 ammonium to nitrate ratio (Gholamnejad et al., 2023; Wu et al., 2024) This spectacle may be due to the dynamic equilibrium formed by the antagonism between NH_4_^+^ and NO_3_^-^ ions to promote the absorption of essential elements, therefore resulting in a significant increment in chlorophyll contents and photosynthetic capacity (**Figure 5**), and enhancing blueberry growth (**Figure 3**). In this experiment, the application of different proportions of ammonium nitrate nitrogen also significantly affected the accumulation of mineral elements in blueberry roots and stems. The N, P, and K contents in blueberry reached the maximum when the NH_4_^+^ and NO_3_^-^ ratios were 25:75 and 50:50, indicating that ammonium/nitrate mixed application could accumulate more nitrogen in plant leaves.

In plants, sugar and sucrose are the final product of photosynthesis, which are initially transported in the phloem to energize cells for the improvement of plant growth and development and activation of the defense system during abiotic stresses (Mehdi et al., 2024). Under abiotic stress conditions, plants need more energy to survive and protect cells from oxidative damage. Thus, sugars are vital sources of energy (Zhang et al., 2022). The results of this study showed that the soluble sugar and starch contents in blueberry leaves increased first and then decreased with the increase of the proportion NH_4_^+^ and NO_3_^-^ (**Figure 8**). These results suggest that appropriate NH_4_^+^ and NO_3_^-^ ratios facilitate the minerals uptake and accumulation (**Figure 7**), thus enhanced photosynthetic capacity and sugar synthesis (Tabatabaei et al., 2008; Alt et al., 2017; Cui et al., 2017; Signore et al., 2020; Morales De Los Ríos et al., 2021; Chen et al., 2024).

To observe the physiological changes of plants during nutritional treatments, the SOD activity and MDA content as essential indicators to reflect plant metabolism and stress resistance (Agarwal et al., 2020; Sathee et al., 2021; Lu et al., 2022). The results showed that the SOD activity of blueberry leaves was the lowest when the N source was only NH_4_ (100:0), and the highest SOD activity was when the N source was only NO_3_ (0:100). In contrast, the MDA contents were maximum in 100:0 and minimum in 0:100 treatments as shown in **Figure 6**, indicating that might be due to increasing of the proportion of NO_3_ in the N source was conducive to improving the ammonium stress of blueberries. In this experiment, applying different proportions of ammonium nitrate nitrogen affected the flowering time and flowering rate of blueberries (**Figure 9**). The plants were the first to enter the flowering stage when the ammonium nitrate ratio was 50:50, indicating that the appropriate ratio of ammonium nitrate nitrogen (50:50 treatment) was conducive to early flowering in blueberry. The higher the NH_4_^+^-N ratio, the faster the flowering speed and shorter the duration, indicating that the plants had a higher demand for NH4^+^-N in the nitrogen source after entering the first flowering stage (**Figures 10**, **11**). In contrast, the high proportion of NO_3_-N significantly reduced the flowering rate of blueberry and delayed flowering initiation, indicating that blueberry was more adaptable to the high proportion of NO_3_-N in the reproductive growth stage (**Figure 11**). In this study, the fruit setting rate of blueberry had the same trend as the duration of flowering, and both of them increased first and then decreased with the decrease of the proportion of ammonium nitrate nitrogen applied, indicating that the appropriate proportion of mixed nitrogen (50:50 treatment) was conducive to improving the fruit setting rate of blueberry.

## 5 Conclusion

Blueberries have different requirements for ammonium and nitrate at various growth stages. The ammonium-to-nitrate ratio of 50:50 is conducive for the growth of shoots and accumulation of mineral elements, thus promoting the formation of flower buds, and fruit setting rate. In contrast, the ammonium-to-nitrate ratio of 100:0~75:25 can promote blueberries to enter the full flowering stage early and accelerate the development process from flower to fruit. In order to achieve the goal of high yield and quality of blueberry cultivated on substrate, reasonable fertilization should be applied according to the needs of different growth and development periods of plants. Considering the growth and nutrient use efficiency of blueberry, the N fertilizer level was reduced based on the improved formula of “Shaoyi Blueberry”, and the nutrient solution formula was formulated with NH_4_^+^-N: NO_3_^−^-N as 50:50, which could maximize the high-quality and efficient production of blueberry. Future experiment is needed to modify substrate and nutritional formulation corelation for enhanced blueberry production.

## Data Availability

The original contributions presented in the study are included in the article/**Supplementary Material**. Further inquiries can be directed to the corresponding author.
