# Supplementary material for: Effects of NH4 +-N: NO3 −-N ratio on growth, nutrient uptake and production of blueberry (Vaccinium spp.) under soilless culture
Source: Front Plant Sci. 2024 Oct 17;15:1438811. doi: 10.3389/fpls.2024.1438811 (PMC11536338; doi:10.3389/fpls.2024.1438811)
Supplement: Supplementary file 1 [file Table1.docx]

Table S1. Trace elements in nutrient solution

| Compound | MnSO_4_·  4H_2_O | | H_3_BO_3_ | CuSO_4_·  5H_2_O | ZnSO_4_·  7H_2_O | (NH_4_)_6_Mo_7_O_12_  ·4H_2_O | Na_2_Fe-EDTA |
| --- | --- | --- | --- | --- | --- | --- | --- |
| Molecular weight | | 223.06 | 61.80 | 249.69 | 287.55 | 1235.86 | 424.10 |
| Concentration  （mg/L） | | 2.13 | 2.86 | 0.08 | 0.22 | 0.02 | 50 |
